# Physicians’ behavior following changes in LDL cholesterol target goals

**DOI:** 10.1186/s13584-015-0016-9

**Published:** 2015-06-01

**Authors:** Shlomo Vinker, Haim Bitterman, Doron Comaneshter, Arnon D Cohen

**Affiliations:** Chief Physician Office, Central Headquarter, Clalit Health Services, Tel Aviv, Israel; Department of Family Medicine, Sackler School of Medicine, Tel Aviv University, Tel Aviv, Israel; Siaal Family Medicine and Primary Care Research Center, Faculty of Health Sciences, Ben-Gurion University of the Negev, Beer-Sheva, Israel

**Keywords:** Quality indicators, Clinical guidelines, Hypercholesterolemia, Statins

## Abstract

**Background:**

In 01/2011 Clalit Health Services (CHS), changed the LDL-Cholesterol target definitions in its quality indicators program, from a universal target to values stratified by risk assessment based on ATP III criteria. The objective of this study is to evaluate the effect of this change on achievement of LDL-C targets and on physicians’ prescriptions of statins.

Study Design: A descriptive study based on administrative dataset 06/2010-06/2012.

**Methods:**

Setting: CHS, The largest health maintenance organization in Israel that insures above 4,000,000 beneficiaries.

Patients: Patients who had been in the same risk group throughout the study period.

Measurements: Attainment of targets for LDL-C and purchases of statins prior to, and following, implementation of the guidelines in the CHS quality indicators program.

**Results:**

433,662 patients remained in the same risk groups throughout the study period; 55.8% were women; the average age was 53.0 ± 10.3 years; 63.9%, 13.4%, and 22.7% were at low, medium, and high risk respectively. After implementation, the proportion of patients reaching LDL-C targets increased in all risk groups: from 58.6% to 61.6%, from 55.1% to 61.1%, and from 44.5% to 49.0%, in low, medium, and high risk groups respectively (p < 0.001). The proportion of patients treated with potent statins increased in all risk groups; from 3.4% to 5.6%, from 6.7% to 10.3%, and from 14.5% to 20.3% respectively (p < 0.001).

**Conclusion:**

The risk stratification approach as a basis for the quality indicators program was implemented and better achievement of target LDL-C levels ensued. We suggest that implementation of quality indicators that are consistent with the current literature can lead to improvements that exceeds temporal trends.

## Background

Healthcare services operate in increasingly complex environments, characterized by growing specialization, fragmentation of medical care, and soaring costs that do not always reflect healthcare value. Healthcare quality assurance programs aim to enhance the extent to which healthcare services achieve desired outcomes, according to the prevailing professional literature. An increasing number of pay-for-performance systems employ financial incentives to achieve quality measures that are focused on health outcomes, rather than on process-of-care [1]. However, the pay-for-performance model has been reported to yield only modest [2] and even negligible and inconsistent improvements in healthcare quality [3,4], and the costs of such programs have yet to be assessed [5].

The National Cholesterol Education Program Adult Treatment Panel III (ATP III) guidelines serves as the benchmark for the assessment of the quality of treatment of hyperlipidemia [6]. Published studies have demonstrated low adherence to them, despite assessment of their relative cost-effectiveness [7]. For example, Barham et al. found that the challenge facing implementation of ATP III guidelines is much greater for intermediate- and high-risk patients than for low-risk patients. [8]. Lee et al. also noted that the rate of achieving target LDL-C levels was lower in patients at higher risk for cardiovascular disease [9]. Computerized clinical decision support systems, including recommendations tailored to patient characteristics, have been shown to mildly increase physician adherence to ATP III guidelines [10], but not to exert a statistically significant effect on LDL-C target achievement [11]. Numerous barriers to guideline adherence by physicians have been identified [12].

All Clalit Health Services (CHS) institutions are required to measure and follow their performance in core clinical areas of operation including both processes of care and healthcare outcomes. At the beginning of every calendar year, CHS publishes its quality indicator program for the upcoming year, with details of targeted changes and the rationale behind them. Guidelines and performance updates are transferred electronically to all CHS physicians and nurses on a monthly basis.

In 2006, CHS set an ambitious target of LDL cholesterol < 100 mg/dl, to be reached by 90% of patients who are after therapeutic cardiac catheterization or coronary artery bypass grafts, 65% of patients with diabetes, and 20% of the remaining individuals with hyperlipidemia. In January 2011, the CHS program of quality indicators was revised and the ATP III guidelines for the management of dyslipidemia of patients without diabetes or active ischemic heart disease were used as a guide for risk stratification [6]. Accordingly, all members of CHS without diabetes or ischemic heart disease were stratified by risk assessment and the automatic reminders for CHS physicians were changed accordingly.

The aim of the current study was to evaluate the longitudinal effect of these changes on achievement of LDL-C targets, and on the delivery of care, as measured by medication purchases.

## Methods

This is a descriptive study based on an administrative dataset of purchases of statins and of attainment of risk stratified targets for LDL-C prior to, and following, implementation of ATP III based guidelines in CHS quality indicators program.

### Data sources

Data were accessed from the CHS data warehouse. CHS is the largest health maintenance organization in Israel, insuring and providing healthcare to more than 50% of Israel’s population (more than 4,000,000 beneficiaries). Every person insured by CHS is under the care of a primary care physician (PCP), either a family physician or a pediatrician. Patients only see the PCP to whom they are assigned (except for when their physician is on vacation, when they are out of town, or when there is urgency and their physician is not available). For each visit to a different PCP, a special administrative certificate of approval is needed and the peer physician is instructed to provide only “first aid”. Hence, provision of primary care in CHS is characterized by a high level of continuity [13].

The CHS information system is comprehensive, comprising socio-demographic data; information on the utilization of healthcare services, drug purchases, laboratory and imaging tests, and a wide-scale registry of chronic diagnoses [14]. The epidemiology unit of the CHS maintains a central comprehensive chronic diseases registry. This registry is continuously updated, based on an algorithm integrating all available data (hospitalization discharge diagnoses, chronic diagnoses in the PCP electronic medical record, laboratory test results, drug purchases and other sources). CHS’s registry of patients with chronic diseases serves as the foundation for calculations of healthcare quality indicators for several chronic conditions such as hypertension, diabetes, and ischemic heart disease.

All community pharmacies operated by CHS are computerized and report to a central data repository. All prescriptions of statins that were filled by CHS members between June 1, 2010 and June 31, 2012 were documented. CHS dispenses medications with nominal and almost equal co-payment, which ensures that all prescriptions are documented and that drug selection is not influenced by financial considerations. The formulary choices offered did not change over the study period, as generic atorvastatin had been introduced on 07/2010 and generic rosuvastatin on 06/2010.

LDL-c tests were performed in the CHS central laboratories, using the same techniques during the study period.

### Patient population

The CHS chronic disease registry identifies people with diabetes and after therapeutic cardiac catheterization or coronary artery bypass grafts, for whom the LDL-C target is <100 mg %. These patients have different quality indicator sets. For all other patients with a diagnosis of hyperlipidemia in CHS registry, cardiovascular risk was assessed based on the ATP III. The following variables were used to assess risk: age and gender, smoking status (patients for whom smoking status was missing were recorded as “non-smokers”), hypertension, cardiovascular diseases (ischemic heart disease without therapeutic cardiac catheterization or coronary artery bypass grafts, peripheral vascular disease, state after cerebrovascular accident), and most recent HDL level (on June 2010 and again on June 2012). We did not have data on family history of cardio-vascular diseases.

We stratified the risk groups according to a modification to the ATP III:

- Patients with 0–1 risk factors were classified as “low risk” with LDL-C target of <160 mg/dl.
- Patients with 2 risk factors were classified as “moderate risk” with LDL-C target of <130 mg/dl.
- Patients with > =3 risk factors or with proven cardiovascular diseases were classified as “high risk” with LDL-C target of <100 mg/dl.

510,166 patients were included in the QI program in 06/2010, of them 76,504 changed risk group during the study period and 433,622 were in the same risk group in 06/2012. Only patients who had been in the same risk group throughout the study period (June 2010 to June 2012) were included in the analysis.

Demographic data included: Age, gender and socio-economic status (SES); low SES was defined as exemption from social security payments.

### Main outcome measures

1. The proportion of patients who reached the LDL-C target value for their risk category.
2. The proportion of patients using potent statins (Atorvastatin and Rosuvastatin) and first- line statins (all other statins) in each risk category.

Outcomes were assessed every six months, starting six months prior to implementation of the stratified approach, and continuing until 18 months after implementation. As it was a retrospective study we evaluated at each point only patients with a valid LDL-C test (a valid test had been defined as a test done in the last 12 months).

The study was approved by Clalit Health Services ethics committee. The committee states that there is no need of informed consent of study subjects.

### Statistical analysis

Comparison between three groups of patients (low, medium, and high risk) with regard to demographic parameters (gender, age, etc.) was performed using one-way analysis of variance (ANOVA) and Chi-square tests, as applicable.

ANOVA with repeated measures (over time) was performed to assess the time trend in the various outcome parameters (prescription of statins, achievement of LDL targets). Contrast analysis was used to compare successive time points vs. baseline.

The statistical significance level was set to 0.05 and the SPSS for Windows software, version 19.0 (Chicago, IL), was used for the analysis.

## Results

The study population included all the 433,362 patients of CHS who remained in the same risk group throughout the study period. Their socio-demographic characteristics are summarized in Table 1. Patients in the low risk category tended to be younger (p < 0.001), and a larger proportion were females (p < 0.001). Patient risk factors are summarized in Table 2.

**Table 1:** **Sociodemographic characteristics of the study population**

	**Total**	**Low risk**	**Moderate risk**	**High risk**	**p value** ^**a**^
Number of individuals	433,662	277,234	58,214	98,214	
% of the cohort	100%	63.9%	13.4%	22.7%	
Age - mean (SD)	53.0 (10.3)	49.2 (12.7)	60.4 (7.8)	59.2 (10.3)	<0.001
Males (%)	44.2%	34.2%	52.3%	78.7%	<0.001
Low socioeconomic status (%)	34.9%	34.8%	32.3%	36.7%	<0.001

^a^For difference between the three groups.

**Table 2:** **Risk factors of participants according to ATPIII**

	**Total**	**Low risk**	**Moderate risk**	**High risk**
Number of individuals	433,662	277,234	58,214	98,214
Male (> = age 45 years)	140,965 (32.5%)	50,264 (18.1%)	29,185 (50.1%)	61,516 (62.6%)
Female (> = age 55 years)	127,264 (29.3%)	76,815 (27.7%)	26,721 (45.9%)	23,728 (24.2%)
Current smoking	76,621	42,933	8,710	24,978
Low HDL cholesterol	56,290	15,650	10,614	30,026
Hypertension	125,128	22,410	45,631	57,087
Ischemic heart disease	59,931	0	0	59,931
State after cerebrovascular accident	21,864	0	0	21,864
Peripheral vascular disease	10,436	0	0	10,436
High HDL cholesterol^a^	80,789	69,236	1,181	10,372

^a^High HDL cholesterol – is a protective factor (in patients with high HDL cholesterol the score of risk factors is subtracted by one).

**Figure 1:**
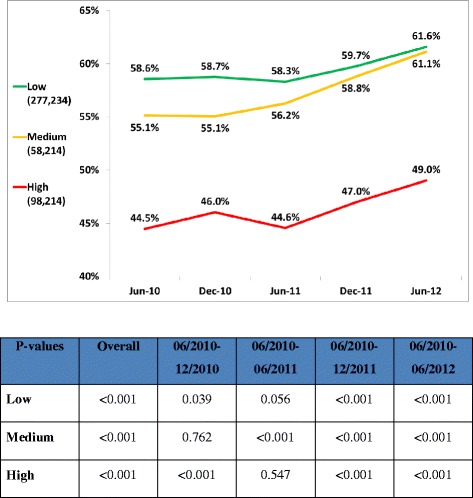
The proportion of patients, stratified by cardiovascular risk who achieved ATP III LDL cholesterol targets during a two-year period (program launched on January 1, 2011).

Figure 1 depicts the trend of target LDL-C achievement according to risk categories. At the beginning of the program target LDL-C achievement was 46.0%, 55.1% and 58.7% in the high, medium and low risk groups respectively. Small changes (and even a decline) were observed in the first six months after launching the program. Changes became more prominent and significant one year after the introduction of the new stratified targets. At the end of 18 months follow-up, target LDL-C achievement was significantly higher: 49.0%, 61.1% and 61.6% in the high, medium and low risk groups, respectively.

**Figure 2:**
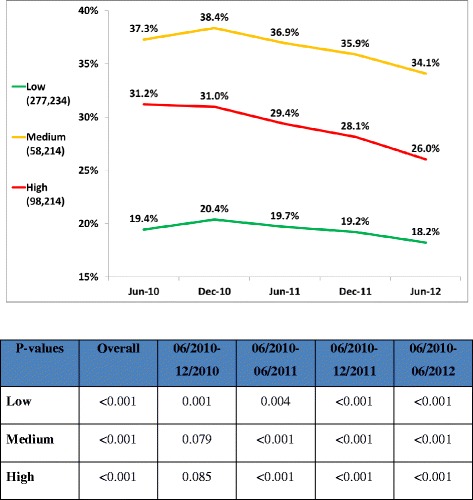
Monthly first line statin prescriptions (program launched on January 1, 2011).

**Figure 3:**
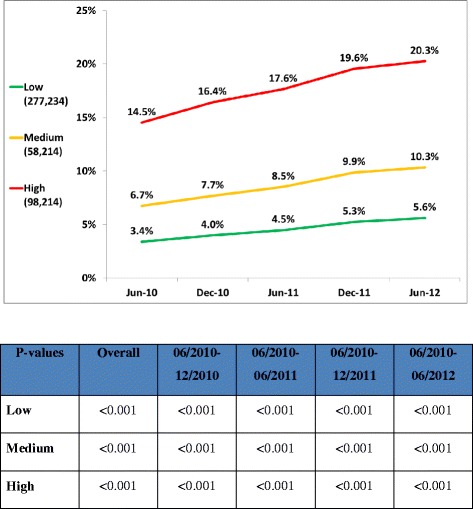
Monthly potent statin prescriptions (program launched on January 1, 2011).

Figures 2 and 3 demonstrate the secular relationship of first line and potent statin utilization, respectively. For all risk categories there was a significant continual decline in the use of first-line statins and a parallel increase in the percent of individuals on potent statins. The prescription of first- line statins declined from 31.0% to 26.0%, from 38.4% to 34.1%, and from 20.4% to 18.2% in the high, medium and low risk groups respectively. The prescription of potent statins increased from 16.4% to 20.3%, from 7.7% to 10.3%, and from 4.0% to 5.6% in the high, medium and low risk groups respectively. The magnitude of the change was greater for higher risk categories. Changes in prescription habits, as reflected by statin purchases, preceded changes in achieving LDL targets.

## Discussion

This study showed that the rate of achievement of target LDL-C decreased during the first 6 months after the implementation of the stratified approach, yet increased throughout the subsequent year. Purchases of first-line statins decreased and purchases of potent statins increased in a complementary manner, starting from six months after implementation of stratified quality indicators based on ATP III guidelines. This was true for individuals in all three cardiovascular risk categories, and continued throughout the 18 month study period. This temporal change in process of care and patient outcomes may be partially explained by the influence of the healthcare policy change on physicians’ clinical behavior [15]. Financial incentives were not awarded, nor immediate health benefit conferred. Thus, physician recognition of the value of quality measures and confidence in their validity might be the prime factors determining their improved adherence.

More than 10% of the total number of beneficiaries of CHS comprised the population of the current study. Treatment of chronic conditions, such as hyperlipidemia, requires committed and persistent long term care of large numbers of patients over the course of years, with relatively high estimations of numbers needed to treat (NNT) to prevent a medical event [16,17]. Since the patients evaluated in the current study had already been diagnosed with hyperlipidemia based on earlier recommendations, CHS guidelines recommended measuring their lipid levels annually. Moreover, physicians received reminders when opening a patient’s computerized file to repeat lipid profile testing if more than a year had passed since previous testing.

The earlier CHS policy of a uniform LDL-C target did not result in improved control of hyperlipidemia, even though identical effective, convenient, medical treatments with minimal side effects were available. Moreover, the goal of reaching this target in 20% of the hyperlipidemic population could even have an opposite effect, with physicians preferring to focus on patients who seemed easier to treat rather than those in most need of treatment. We found differences in the use of first-line statins, and particularly in the use of potent statins, among patients with different risk levels, even before implementation of the ATP III guideline- based quality indicators. Such deviation from the uniform target established in CHS during that period attests to differences in clinical decision making when targets were not based on established clinical guidelines published in the medical literature. Ongoing updating of quality indicators and clinical targets according to current scientific knowledge is thus especially important, as is assessment of the benefits expected in physician behavior [18].

We observed a lag of six months in the improvement in patients’ lipid profiles after the implementation of the new quality indicators. Possible reasons for the time lapse in perceived improvement were: the large population involved, which in some cases comprised up to 15% of a physician’s patients list, and the time required for a physician to identify relevant patients, to evaluate their willingness to a change in treatment, to enact the change, and to conduct a follow-up blood test to verify its effect.

Even at the end of the study period, fewer than half (46.3%) of the individuals classified with high cardiovascular risk purchased statins. Similarly, less than half (49%) reached the LDL-C target. Non-interventional studies have shown comparable rates for both statin use and LDL-C target achievement among high risk patients. Data from the United States National Health and Nutritional Examination Survey (NHANES) showed 54.4% of individuals with diabetes to achieve LDL-C < 100 mg/dL in 2007–2008 [19]. In a multi-center study of diabetic patients in Korea, only 47% reached ATP III targets for LDL-C, even though 96% were taking statins, yet physicians perceived that 71% achieved the targets [20]. Other Israeli studies found that achievement of LDL-C targets among patients with hyperlipidemia with diabetes or established cardio-vascular diseases is also sub-optimal [21-24]. Achieving LDL-C levels of less than 100 mg/dl were 65-67% among patients with coronary disease [21,23,24], 57% in diabetic patients [23] and 46.7% among patients with peripheral vascular disease [24].

Underutilization of lipid-lowering drugs among individuals with diabetes has also been reported in studies conducted in Germany [25] and in China [26]. In a study of individuals who were referred to a lipid clinic, LDL-C targets were reached by only 20% and 45% of those not treated and treated with statins, respectively [27].

18 months after change in quality indicators definitions 3%, 6% and 2.9% more of the hyperlipidemia patients with high, medium and low risk respectively achieved target LDL-C levels. It means additional 1,500, 1,800 and 2,900 patients at the high, medium and low risk groups respectively. Published literature suggests that the effect observed is clinically significant and has the potential to result in a significant reduction in major coronary events [28,29].

A main strength of the current study is the two year follow-up of all patients classified in the same risk categories in a large health maintenance organization. This precludes the selection bias confronting prospective studies that include only physicians and clinics who express willingness to participate [10,11]. The current study also contrasts with investigations that assessed target achievement as reduction in cholesterol, without stratification by risk groups [30]. The comprehensive and valid data warehouse of CHS, including drug purchases and laboratory tests is another strength of the study. Perhaps most important, we demonstrated a static situation before the declared implementation of ATP III guideline quality indicators, changes in statin use following the policy change, and increased achievement of LDL-C targets subsequent to the changes in statin use. Still, the study design does not afford conclusions regarding causality; and neither individual patient changes nor adherence of physicians to ATP III guidelines were assessed. Nevertheless, the temporal change observed, first in increased purchases of potent statins, and then in increased rate of LDL-C target achievement, suggests a relationship between these outcomes.

This study has a number of additional limitations. We stratified the patients to three risk groups according to the ATP III guidelines. We were unable to retrieve family history of ischemic heart disease or to follow the risk classification calculation on the entire hyperlipidemic population of the CHS so there may be some misclassification in our cohort. But misclassification would be expected to be mainly in the direction of putting patients with high risk into a lower risk group, so the thrust of the PCPs in the program would not be affected. We cannot refute the possibility that factors other than those investigated may have affected clinical decision making and outcomes. No changes in approvals or co-payments of relevant drugs occurred during the study period in the face of the expiration of patents on Atorvastatin and Rosuvastatin in Israel at the beginning of the study period. However, it may be that approval policy of potent statins had become more liberal even before the study period, pending the introduction of generics.

We presume that changes in the National Quality Indicators initiative at that period did not have any effect on PCPs behavior in CHS, as treatment goals in the CHS were more ambitious throughout the study period. There were no results of large relevant studies or essentially different clinical guidelines published that could be expected to affect the behavior of the physicians or the general population. The only developments were the notification of physicians of the change in CHS quality indicators regarding treatment of hyperlipidemia, and updates in the reminders in the personal computerized file of patients consistent with the policy change.

## Conclusions

In conclusion, we found that a change in the definition of the quality indicators for the treatment of hyperlipidemia was associated with a change in physician behavior and with improvement in the rate of achievement of target values within 18 months of implementation. The findings suggest that implementation of a quality measure that is tied to established and accepted clinical guidelines, in a setting where physician results are tracked, has the potential to motivate physician behavior and achieve improved clinical results (beyond temporal trends), even in the absence of direct “pay for performance” incentives.

## Abbreviations

ATP III: Adult Treatment Panel III
CHS: Clalit Health Services
HDL: High density lipoproteins
LDL-C: Low density lipoprotein cholesterol
PCP: Primary care physician
NNT: Numbers needed to treat
NHANES: National Health and Nutritional Examination Survey
